# Unusual nail pigmentation following cyclophosphamide-containing chemotherapy regimen

**DOI:** 10.4103/0253-7613.68433

**Published:** 2010-08

**Authors:** Santosh Kumar, Rakesh Dixit, Saurabh Karmakar, Sayan Paul

**Affiliations:** Department of Pulmonary Medicine, Chhatrapati Shahuji Maharaj Medical University (erstwhile King George Medical University), Lucknow, Uttar Pradesh, India; 1 Department of Pharmacology and Therapeutics, Chhatrapati Shahuji Maharaj Medical University (erstwhile King George Medical University), Lucknow, Uttar Pradesh, India; 2 Department of Radiotherapy, Chhatrapati Shahuji Maharaj Medical University (erstwhile King George Medical University), Lucknow, Uttar Pradesh, India

**Keywords:** Chemotherapy, cyclophosphamide, nail pigmentation

## Abstract

Cyclophosphamide therapy may rarely cause pigmentation of the nails which is of different patterns. We report a patient who developed pigmentation of nails after six cycles of cyclophosphamide, methotrexate, and 5-flourouracil chemotherapy, each repeated after 28 days for breast cancer. The patient developed nail pigmentation that started proximally and spread distally and involved all the nails of both hands and feet except the second and third toenails of right foot. Using Naranjo ADR Probability Scale, the case revealed a “probable” association with cyclophosphamide.

## Introduction

Cyclophosphamide (cytophosphane), a chemotherapeutic agent belongs to the nitrogen mustard group of alkylating agents. It acts after being metabolized in the liver to the active metabolite phosphoramide, which alkylates DNA and inhibits replication.[1] Apart from systemic toxicity such as anaphylaxis, bone marrow suppression, and hemorrhagic cystitis, cyclophosphamide can cause a variety of mucocutaneous side effects including anagen effluvium, stomatitis, and hyperpigmentation involving the skin, mucous membranes, nails, palms, and soles and teeth.[2] Chemotherapy-induced nail pigmentation in patients with skin type V is not an uncommon event, which is probably underestimated and under-reported.[3] Clinical presentation varies, depending on which nail structure is affected and the severity of the insult.[4] We report the case of a patient who developed an unusual pattern of nail pigmentation following chemotherapy for breast cancer.

## Case Report

A 60-year-old lady was referred to our center with a complaint of progressively increasing breathlessness and cough off and on since 2 months. She had no past history of respiratory complaints. Her general examination revealed a right sided breast lump, which was firm and non-mobile.

Her treatment history revealed that she had noticed a breast lump about 10 months back, the size of which was about 2 × 2 cm. She had waited 2 months before seeking proper specialist medical care. Histopathology and immunohistochemistry examination was done by the referring center which revealed the lump to be hormone receptor negative Her-2/ neu positive ductal carcinoma *in situ* with node negative status. She was prescribed on neoadjuvant chemotherapy in the form of cyclophosphamide, methotrexate, and 5-flourouracil for six cycles by the previous center.

She had taken oral cyclophosphamide 150 mg daily for 14 days with injection methotrexate (40 mg/m^2^) and injection 5-flourouracil (600 mg/m^2^) on days 1 and 8, repeated every 28 days. She had completed six cycles by the time she presented to our center. She had been administered a total of 12.6 g cyclophosphamide, 720 mg methotrexate, and 11 gm of 5-florouracil.

**Figure 1:**
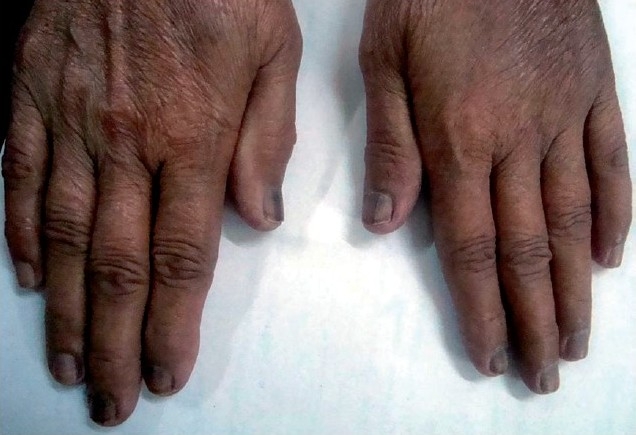
Nails of both hands pigmented more deeply than the toenails.

**Figure 2:**
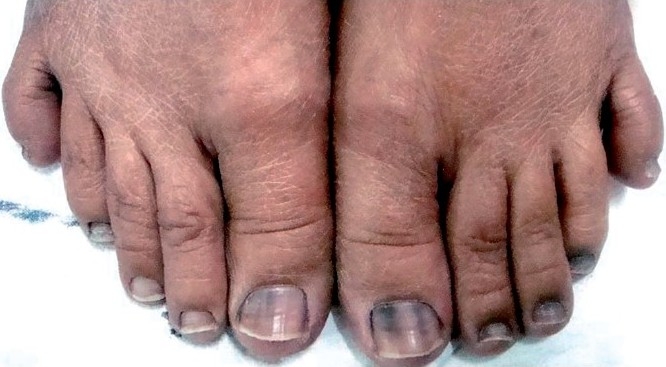
Pigmentation of nails of both feet with sparing of the nails of the IInd and IIIrd toe of the right foot.

The patient stated that she developed nail pigmentation which started proximally and spread distally over the duration of chemotherapy. Pigmentation involved all the nails of the hand and toes except the second and third toes of the right foot [Figures 1 and 2]. The pigmentation pattern was uniform in all the nails of the hands and toes except first toe of both feet where it was in form of transverse bands at presentation. The pigmentation varied from black to brown and was deeper and extensive in the finger nails compared to the toe nails. The patient had noticed progressively increasing pigmentation from the third cycle of chemotherapy onward and did not regress after chemotherapy had been stopped. There was no associated skin or mucous membrane pigmentation. The patient did not suffer significant systemic toxicity.

A chest x-ray of the patient revealed a right-sided pleural effusion. The pleural fluid was hemorrhagic on gross examination and cytochemical analysis revealed a lymphocyte predominant nature with adenosine deaminase level of 18U/L. On microscocopic examination of the pleural fluid sediment, malignant cells with a high nucleo-cytoplasmic ratio were found. Because of the advanced nature of the disease and the exhaustive past treatment, the patient refused any further workup. She was subsequently lost to follow-up.

## Discussion

Nail pigmentation is caused by chemotherapeutic agents like cyclophosphamide, doxorubicin, hydroxyurea, and bleomycin. Cyclophosphamide has been reported to cause diffuse black pigmentation, slate grey to black longitudinal streaks, or diffuse dark-grey pigmentation located proximally with overlying black transverse bands.[3] Other findings are onychodystrophy, onycholysis, Beau’s lines, and Muehrcke lines.[4]

Toxicity may be asymptomatic and limited to cosmetic concerns; however, more severe effects, involving pain and discomfort can occur. Taxanes and anthracyclines are the antineoplastic drug groups most commonly implicated. It is suggested that the administration schedule may influence the incidence of nail abnormalities. Before instituting chemotherapy, patients should be educated regarding potential nail toxicities and strategies for prevention implemented.[5]

We attributed the extensive black pigmentation of nails to cyclophosphamide because 5-fluorouracil only causes proximal pigmentation and methotrexate is known to cause golden pigmentation.[6] Thus cyclophosphamide-induced nail pigmentation may have varied patterns as was noted in our patient who had pigmentation of the nails of hands and toes except the second and third toe of right foot. The pigmentation was deeper and extensive in the hand nails (fingernails) compared to the toe nails. Extensive search of the literature revealed only one reported instance of cyclophosphamide-induced pigmentation involving the nails of the thumb, index finger, and half of middle finger of both hands which is very much different from the pattern seen in our case.[7] Various mechanisms proposed for the pathogenesis of cyclophosphamide-induced nail pigmentation including genetic predisposition, toxic effect of the drug on the nail bed and matrix, photosensitization, and focal stimulation of melanocytes in the matrix.[8]

Using the Naranjo ADR Probability Score,[9] it was concluded that there was a probable association with cyclophosphamide. We are reporting this case to increase awareness among prescribers about this uncommon side effect of a very common chemotherapeutic agent in the Indian scenario.
